# The first record of *Tremoctopus violaceussensu stricto* Delle Chiaje,1830 in southwestern Gulf of Mexico gives a hint of the taxonomic status of *Tremoctopus gracilis*

**DOI:** 10.3897/zookeys.1012.55718

**Published:** 2021-01-26

**Authors:** María de Lourdes Jiménez-Badillo, César Meiners-Mandujano, Gabriela Galindo-Cortes, Piedad S. Morillo-Velarde, Roberto González-Gómez, Irene de los Angeles Barriga-Sosa, Ricardo Pliego-Cárdenas

**Affiliations:** 1 Laboratorio de Biología Pesquera y Acuicultura. Instituto de Ciencias Marinas y Pesquerías, Universidad Veracruzana. Hidalgo 617. Col. Río Jamapa. Boca del Río, Veracruz, México. C.P. 94290, Mexico; 2 CONACYT- Instituto de Ciencias Marinas y Pesquerías, Universidad Veracruzana. Hidalgo 617. Col. Río Jamapa. Boca del Río, Veracruz, México. C.P. 94290, Mexico; 3 Posgrado en Ecología y Pesquerías, Universidad Veracruzana. Mar Mediterráneo 314. Fracc. Costa Verde. Boca del Río, Veracruz, México. C.P. 94290, Mexico; 4 Laboratorio de Genética y Biología Molecular, Planta Experimental de Producción Acuícola, Universidad Autónoma Metropolitana Unidad Iztapalapa. Av. San Rafael Atlixco 186. Col. Vicentina. Iztapalapa, Cd. de México. C.P. 09340, Mexico; 5 Departamento de Biología Comparada, Facultad de Ciencias, Universidad Nacional Autónoma de México, Av. Universidad 3000, Ciudad Universitaria, Delegación Coyoacán, C. P. 04510, Mexico

**Keywords:** Blanket octopus, genetic divergence, geographic distribution, new record, range extension Mexico, Veracruz Reef System, 16S haplotype

## Abstract

Knowledge on species taxonomic identity is essential to understand biological and biogeographical processes and for studies on biodiversity. Species the genus *Tremoctopus* have been confused in the past and are inconsistently identified. To clarify of the taxonomic diagnosis *Tremoctopus violaceus* Delle Chiaje, 1830, an evaluation of morphological and meristic characters, as well as morphometric indices and genetic analyses, was undertaken. The analyzed octopod was an opportunistically collected mature female of 640 mm in total length, with a mantle length of 135 mm and a total weight of 1.02 kg. Evidence of autotomy as a defensive mechanism for protecting the egg mass is presented. The 16S haplotype sequenced from this specimen represents the first one publicly available for this species from the Gulf of Mexico. The genetic divergence between this haplotype and those reported from the Pacific Ocean is representative of interspecific variation in other taxa, which suggests that “*T. violaceus*” in the Pacific Ocean (KY649286, MN435565, and AJ252767) should be addressed as *T. gracilis* instead. Genetic evidence to separate *T. violaceus* and *T. gracilis* is presented. The studied specimen from the Gulf of Mexico represents the westernmost known occurrence of *T. violaceus* and the first record from the southwestern Gulf of Mexico.

## Introduction

Tremoctopodidae is one of the four families within the superfamily Argonautoidea Cantraine, 1841 (Mollusca, Cephalopoda), all of which are characterized by marked sexual size dimorphism, with small or dwarf males and larger females, some of which reach 2 m long (Naef 1923; Norman 2000). Such extreme dimorphism is not seen in any other animal group (Norman et al. 2002). The Tremoctopodidae is represented by a single genus, *Tremoctopus* (blanket octopus), with four species currently recognized as valid: *Tremoctopus gelatus* Thomas, 1977 which is meso-bathypelagic, gelatinous, with circumtropical and temperate distribution; *Tremoctopus robsoni* Kirk, 1884 which was described from waters off New Zealand; *Tremoctopus gracilis* (Eydoux & Souleyet, 1852) which occurs in the Pacific and Indian oceans; and *Tremoctopus violaceus* Delle Chiaje, 1830 which is an epipelagic (1–250 m depth), muscular, heavily pigmented, and restricted from 40°N to 35°S in the Atlantic Ocean, including the Gulf of Mexico, Caribbean Sea, and Mediterranean Sea (Voss 1967; Thomas 1977; O’Shea 1999; Quetglas et al. 2013; Mangold et al. 2018).

The most comprehensive systematic review of Tremoctopidae was by Thomas (1977). Based on the morphological characteristics of the hectocotylus, he proposed two subspecies for *T. violaceus*: *T. v. violaceus* from the Atlantic and *T. v. gracilis* from the Indo-Pacific. More than two decades later they were reclassified as species using the same morphological considerations (Mangold et al. 2018). However, the difficulty in separating these taxa based solely on male morphology, as well as the absence of molecular phylogenetic analyses of the genus, has caused taxonomic confusion. This is evident in occurrence records of these two species that lie outside the geographical limits indicated by Thomas (1977) and Mangold et al. (2018). Examples of such cases are records published by Zeidler (1989), García-Domínguez and Castro-Aguirre (1991), Norman et al. (2002), Chesalin and Zuyev (2002), Nabhitabhata et al. (2009), Chiu et al. (2018), and many historical records in the Ocean Biogeographic Information System (OBIS 2020).

Specimens of *T. violaceus* and *T. gracilis* are relatively rare in catches and, therefore, remain poorly known, despite their sporadic appearance since 1914 (OBIS 2020). Information on *T. violaceus* has been obtained from three sources: 1) occasional encounters of living or dead individuals (Voss 1956; Thomas 1977; Lozano-Soldevilla 1991; Chesalin and Zuyev 2002; Díaz and Gracia 2004; Nabhitabhata et al. 2009; Almeida-Tubino et al. 2010; Quetglas et al. 2013), 2) collections or captures (Salisbury 1953; Thomas 1977; Arocha and Urosa 1983; Nesis 1987; Biagi and Bertozzi 1992; Judkins et al. 2017) and 3) as remains in stomach contents of large pelagic fishes (Bello 1993; Almeida-Tubino et al. 2010). Causes of the taxonomic uncertainty of these species are the intrinsic limitations in obtaining specimens, the difficulties in distinguishing morphologically similar species, and the limited number of genetic sequences currently available in GenBank for *T. violaceus*.

Therefore, the addition of sporadic findings of *Tremoctopus* species, like in the present study, is of utmost importance for the taxonomic clarification of the genus. Hence, this study reports the first record of *Tremoctopus violaceussensu stricto* in the southwestern Gulf of Mexico, supported by an integrative taxonomic approach that includes both morphological and genetic analyses. This study also establishes the genetic baseline to resolve the phylogenetic relationships between *T. violaceus* and *T. gracilis*.

## Methods

**Figure 1.**
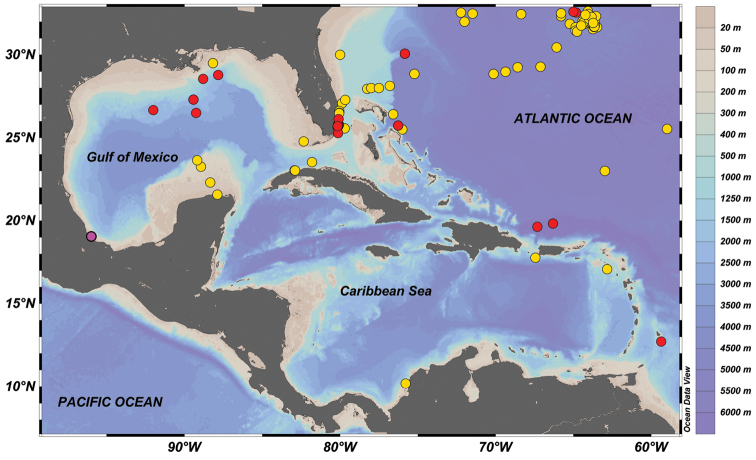
Observed distribution of *Tremoctopus violaceus* in Gulf of Mexico and adjacent areas based on Thomas (1977) (red dots), records contained in OBIS (2020) data base (yellow dots), and the new record from the southwestern Gulf of Mexico (present study; pink dot). Map prepared using Ocean Data View software (Schlitzer 2016).

The studied specimen was found alive by fishermen in the Veracruz Reef System, at the fishing harbor of the town of Antón Lizardo (19°03'24"N, 95°59'17"W), Veracruz state, in the southwestern Gulf of Mexico (Fig. 1) on 20 July 2019 at approximately 13:00 hrs. The body condition, live coloration, and behavior were recorded *in situ*, then the specimen was preserved on ice and sent to the Laboratorio de Biología Pesquera y Acuicultura, Instituto de Ciencias Marinas y Pesquerías (Universidad Veracruzana) for study.

In the laboratory, a photographic record of the fresh octopod was obtained and the species was determined following Voss (1956), Thomas (1977), Roper and Voss (1983), and Mangold et al. (2018). The terminology and measurements used follows Thomas (1977) and Finn (2013). All measurements were made when the specimen was fresh and are given in millimeters; the total weight is in kilograms. The morphological indices are expressed as a percentage of the dorsal mantle length. The number of gill filaments and nuchal folds were also recorded.

Given the good condition of the specimen and to keep it intact, no internal organs were removed for analysis. Muscle tissue samples from the mantle and arm were taken for genetic analysis. Tissues were preserved in 95% ethanol and maintained at –4 °C for 72 h before processing for DNA extraction, following the procedure suggested by Wall et al. (2014). The specimen was fixed in 10% formalin, transferred to 75% ethyl alcohol, and deposited in the Colección Nacional de Moluscos, Universidad Nacional Autónoma de México (Mexico City) under the voucher number CNMO 8042.

The genetic analysis was conducted at the Laboratorio de Genética y Biología Molecular, Planta Experimental de Producción Acuícola, Universidad Autónoma Metropolitana Iztapalapa. Total DNA was extracted using the Wizard Genomic DNA Purification Kit (Promega). DNA amplification was carried out through a polymerase chain reaction (PCR) using the ribosomal 16S primers from Simon et al. (1991). The mitochondrial fragment 16S is an effective DNA barcode marker for identifying cephalopod species (Dai et al. 2012; Pliego-Cárdenas et al. 2014, 2016; Flores-Valle et al. 2018). PCR conditions and sequencing are as in González-Gómez et al. (2018). The genetic analysis consisted of 1) identifying sequence homology in GenBank (NCBI) using the mega blast algorithm in Blast tool and 2) the phylogenetic inference analysis using the maximum likelihood (ML) method and the GTR+I+G model resolved by JModeltest (Darriba et al. 2012) in RaxMLGUI v. 1.5 (Silvestro and Michalak 2012). Branch support was assessed using 1000 bootstrap (bs) pseudo replicates under the rapid bootstrap algorithm.

Genetic divergences between the sequence obtained in this study and those that were the most similar according to Blast search from GenBank, were calculated in MEGA 7 (Kumar et al. 2016) using the Kimura two-parameter model (K2P). The following homologous sequences from genbank were used: *T. violaceus* (KY649286, MN435565, AJ252767), *Ocythoe tuberculata* Rafinesque, 1814 (GU288520), and *Haliphron atlanticus* Steenstrup, 1861 (AY616971). *Argonauta nodosus* Lightfoot, 1786 (AY545104), *A. hians* Lightfoot, 1786 (KY649285), and *A. argo* Linnaeus, 1758 (AB191108) were used as outgroups, based on the previous study of the phylogeny of Argonautoidea (Strugnell and Allcock 2010).

The results of the genetic analyses are discussed in context to the known distribution of Thomas’s (1977) understanding of *T. violaceus* and *T. gracilis* as subspecies, and to the available records in OBIS (2020).

## Results

**Superfamily Argonautoidea**

**Family Tremoctopodidae**

**Genus *Tremoctopus***

### Tremoctopus violaceus

Taxon classificationAnimaliaOctopodaTremoctopodidae

delle Chiaje, 1830

CEAEE406-6952-5B14-B458-598AB8136E55

Figures 2 , 3

#### Material examined.

Mexico • 1 female, 640 mm TL; 135 mm ML; southwestern Gulf of Mexico, Veracruz, Antón Lizardo; 19°03'24"N, 95°59'17"W; 20 July 2019; Jiménez-Badillo, L; recovered alive by fishermen; GenBank: MT271737; specimen code CNMO 8042.

**Figure 2.**
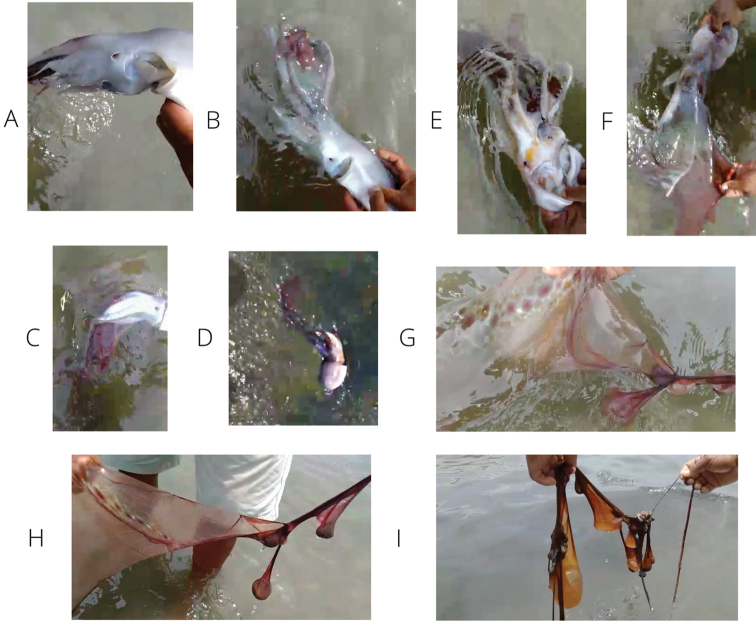
Photographic record of the *Tremoctopus violaceus* specimen (135 mm ML) in natural environment highlighting relevant characters for its taxonomic determination **A** ventral water pore **B–D** web of dorsal arms coiled on the ventral side and deployed when female was feeling threatened **E** egg mass **F** web displaying an iridescent greenish glow and a reddish brown color **G–I** evidence of autotomy: segments detached from interbrachial membrane showing slender arm, part of the connective tissue, circulatory system and chromatophores pattern characteristic of the species.

The analyzed octopod was an adult female (TL of 640 mm, ML_d_ 135 mm, and TW 1.02 kg). It was found alive and was showing signs of disorientation and gross color pattern changes on the blanket from iridescent transparent to reddish-brown (Fig. 2A, D, F). The specimen had no apparent damage. Upon approach and handling by the fisherman, the octopus became threatened, extended her web, and jettisoned her eggs (Fig. 2B–D. A few meters away from the octopus, there appeared what was probably the eggs attached to a rod-like structure, but this could not be collected only recorded by video. This observation provides evidence of autotomy as a means of protection of the egg mass (Fig. 2G–I). The specimen is inferred to be sexually mature (Fig. 2E). Water pores, the coiled web on the ventral side of the animal, and some chromatophores on the web, which are characteristic of the species, were seen and recorded by video (Fig. 2A–D, G).

**Figure 3.**
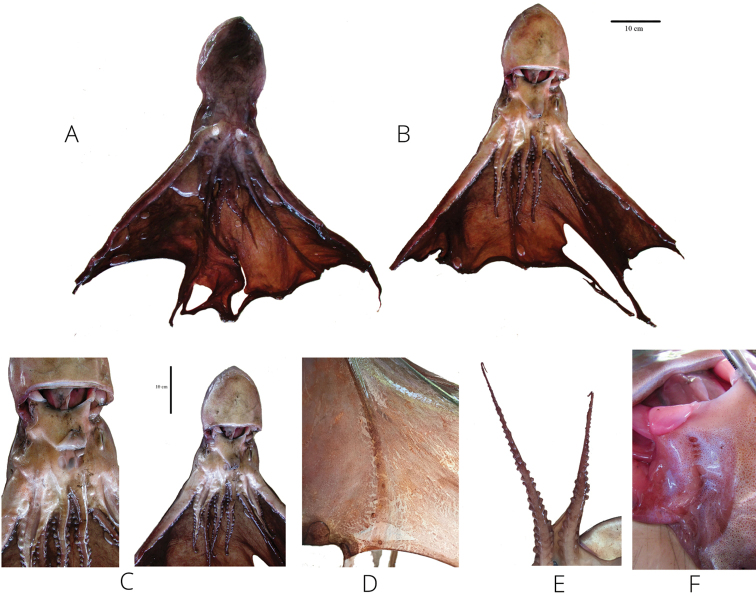
Photographic record of the *Tremoctopus violaceus* fresh specimen (135 mm ML) highlighting relevant characters for its taxonomic determination **A, B** dorsal and ventral view; arms unequal in length; one web between the four dorsal arms; two pairs of cephalic water pores, one pair located on dorsal surface of the head, slightly anterior to eyes at the base of first arms **C** second pair located ventrally, adjacent to funnel opening, at base of fourth arms; eyes large, laterally directed; funnel extends beyond eye level, distal one quarter free **D** bioluminescent tissue **E** biserial suckers on arms decreasing in size towards the distal portion **F** nuchal folds. To see the character dimensions, see Table 1. Scale bars: 10 cm (**A–C**).

**Figure 4.**
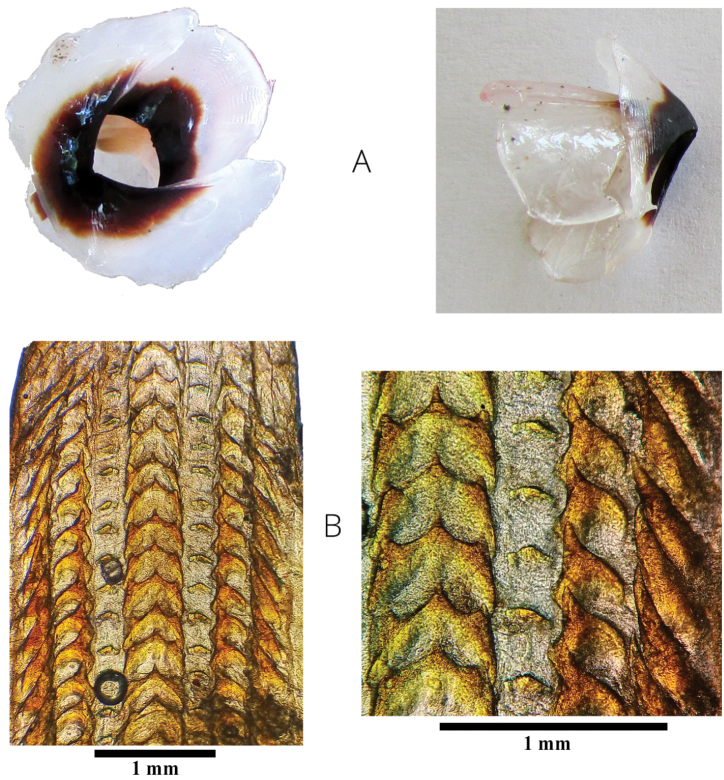
Photographic record of **A** upper and lower beak **B** radula, seven teeth and two marginal plates per transverse row are appreciated. On the approach (bottom right) rachidian teeth tricuspid with an A2 seriation is observed. For beak dimensions see Table 1. Scale bars:1 mm (**B**).

The fresh octopus had a brownish-purple color on the dorsal mantle and the head, while the ventral mantle was iridescent-silvery. The mantle was thick and muscular. The eyes were lateral. It had one pair of cephalic pores on the dorsal head between the eyes, and another, smaller pair on the ventral head adjacent to the funnel opening. The funnel extended beyond eye level and 14 gill filaments were counted. The arms were unequal in length and shape. The dorsal arms (arm pairs I and II) were much longer than the ventral arms (arm pairs III and IV); arms I and II were truncated. The suckers were biserial, decreasing in size towards the distal portion of each arm. One deep web was present between the four dorsal arms. The depth of the interdigital membrane was well developed and V-shaped. The nuchal folds numbered eight (Fig. 3A–E). The radula had seven teeth as well as two thin, rectangular marginal plates per transverse row. The rachidian teeth were tricuspid with an A2 seriation. The first lateral teeth were much smaller than the second lateral and rachidian teeth. The marginal teeth were long and slender and spine-shaped (Fig. 4). The color pattern and the morphological features described above as well as the body measurements and morphometric indices presented in Tables 1, 2 of the analyzed specimen fully correspond to *T. violaceus*, (Thomas 1977; Roper and Voss 1983; Orsi 2009; Mangold et al. 2018).

**Table 1.** Body measurements (in mm) of the *Tremoctopus violaceus* specimen found in the southwestern Gulf of Mexico.

Character	CNMO 8042 specimen
Total length (TL)	640
Dorsal mantle length (ML_d)_	135
Ventral mantle length (ML_v)_	83
Mantle width (MW)	72
Head length (HL)	100
Head width (HW)	94
Arm length I (AL I) (left/rigth)	365*/330*
Arm length II (AL II) (left/rigth)	332*/473
Arm length III (AL III) (left/rigth)	161/162
Arm length IV (AL IV) (left/rigth)	152/179
Web depth interdigital A (WDI A)	Until tip of truncated arm
Web depth interdigital B (WDI B)	Until tip arm
Web depth interdigital C (WDI C)	78
Web depth interdigital D (WDI D)	65
Web depth interdigital E (WDI E)	54
Funnel length (FuL)	58
Free funnel length (FFL)	20
Funnel width (FW) at opening	25
Pallial aperture (PA)	89
Eye diameter (ED)	25
Pore size ventral (PS_v_) (left/rigth)	16×11 / 16×13
Pore size dorsal (PS_d_) (left/rigth)	27×18 / 27×17
Upper beak	
Hood length (HoL)	11.0
Beak height (BH)	17.8
Beak length (BL)	16.1
Beak width (BW)	16.5
Lower beak	
Rostral length (RL)	12.2
Wing length (WL)	18.0
Wing width (WW)	9.5
Beak height (BH)	5.7
Beak length (BL)	14.0
Beak width (BW)	19.1

* Truncated

**Figure 5.**
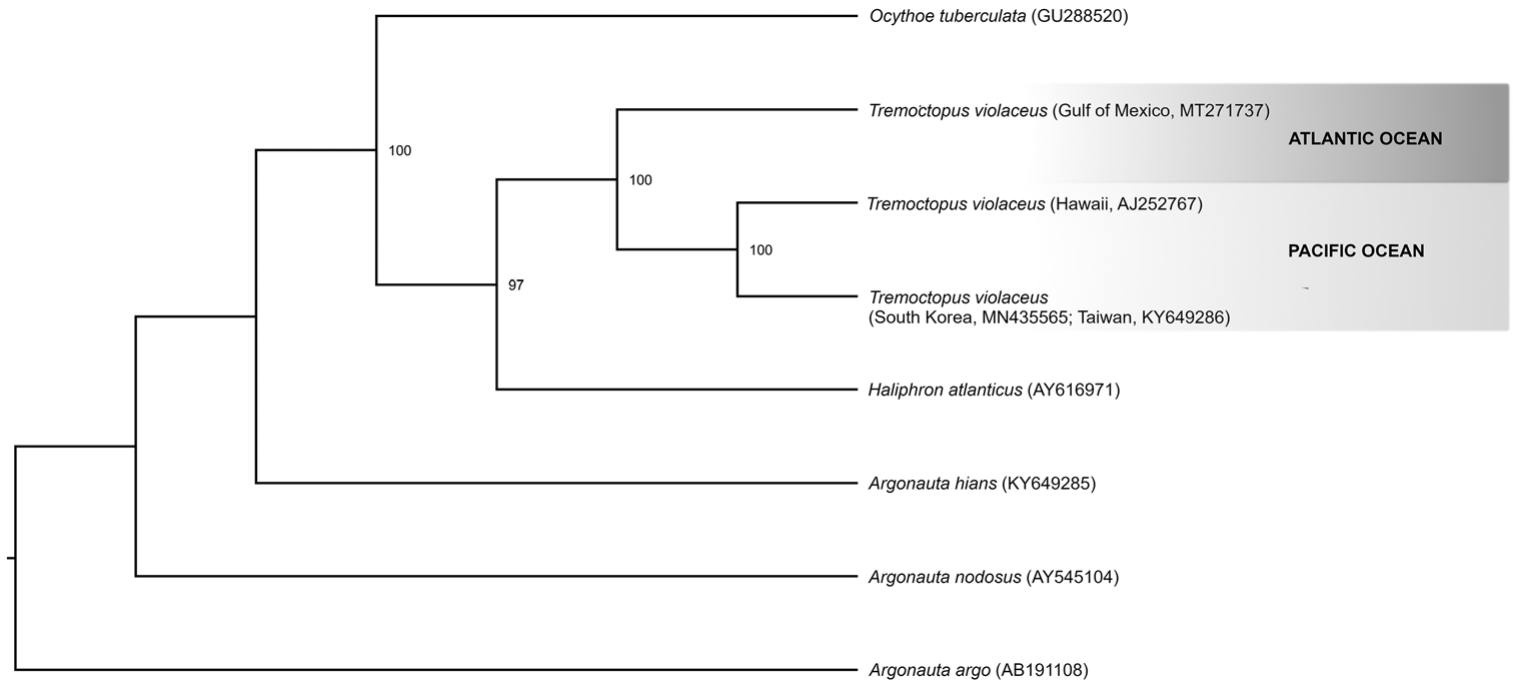
Maximum likelihood phylogenetic tree based on 16S sequences showing the relationships of *Tremoctopus violaceus*. Only bootstrap values above 90 are shown. Records from the Hawaiian Islands and South Korea–Japan were likely misidentified and correspond to *T. gracilis*.

The compiled sequence of the mtDNA16S region (470 bp) obtained in this study (GenBank accession number MT271737) shows over 90% similarities to the *T. violaceus* homologue sequences from South Korea (MN435565), Taiwan (KY649286; Chiu et al. 2018), and Hawaii (AJ252767). This is the first mtDNA 16S haplotype publicly available in GenBank of *T. violaceus* from the Gulf of Mexico. Other public sequences for the species correspond to haplotypes of cytochrome c oxidase subunits I (COI) and III (COIII) genes (AF377978 and GU288522, respectively), and the voucher UMML:31.312. The genetic divergence among the Gulf of Mexico (Atlantic Ocean) specimen and the reference sequences from the Pacific Ocean is 6%, with 31 variable sites. All the *T. violaceus* 16S sequences are clustered in a well-supported monophyletic clade (bs = 100) (Fig. 5); however, the Atlantic Ocean specimen is in a separate clade from specimens from the Pacific Ocean.

**Table 2.** Morphometric indices of the *Tremoctopus violaceus* specimen found in the southwestern Gulf of Mexico.

**Index**	**CNMO 8042 specimen**
Pore length index dorsal (PLI_d_)	20
Pore length index ventral (PLI_v_)	12
Mantle width index (MWI)	53
Head width index (HWI)	70
Mantle arm index (MAI)	41
Arm length index I (ALI I) (left/rigth)	270 / 244
Arm length index II (ALI II) (left/rigth)	246 / 350
Arm length index III (ALI III) (left/rigth)	119 / 120
Arm length index IV (ALI IV) (left/rigth)	112 / 133
Arm formula (AF)	2,1, 4, 3
Arm width index (AWI)	19.26
Free funnel length index (FFuLI)	14.8
Funnel length index (FuLI)	42.9
Head length index (HLI)	74
Mantle width index (MWI)	53.3
Pallial aperture index (PAI)	65.9

## Discussion

The octopus found in the southwestern Gulf of Mexico was a mature female belonging to the species *Tremoctopus violaceus* according to the morphometric, genetic, and biogeographic evidence, this identification is supported by the following features: color pattern, dorsal arms linked by a deep and broad web, arms proportions, sucker position, presence of conspicuous cephalic water pores, extended funnel, counts of gill filaments, morphology of radular teeth, and eggs carried in arms (Voss 1956; Thomas 1977; Roper and Voss 1983; Orsi-Relini 2009; Mangold et al. 2018).

The studied specimen was found to be an adult female. Thomas (1977) indicated that the mantle shape depends on the size of the animals. Isometric growth of the mantle occurs in adults with a mantle length of 100–250 mm and not in juveniles. In this study, the mantle width index (MWI) was 53, which reflects a proportional growth between mantle width and mantle length. On the other hand, in adults, the mantle length continues to increase slightly faster than the head width, which is confirmed in the studied specimen by the head width index (HWI 70). In adult females, the funnel forms a broad transverse band with thin folds of glandular tissue. The funnel is moderate in size, extending beyond the level of the eyes and is free for about a quarter of its length. In the studied specimen, the funnel length (FuL) was 58 mm and the free funnel length (FFL) was 20 mm, almost a quarter of the FuL.

In this species, the dorsal pores (PS_d_) are usually larger than the ventral pores (PS_v_), this was confirmed by 27×18 / 27×17 mm (left/right) vs 16×11 / 16×13 mm (left/right), respectively. The length of arms I and II is at least twice the mantle length, while the length of arms III and IV exceeds the mantle length by about 24 units. The arm formula (AF) 2, 1, 4, 3 agrees with that reported by Guerra (1992), Thomas (1977), and Finn (2014).

Autotomy was observed as a defense mechanism when the female felt threatened. Mangold et al. (2018) remarked that the web is only extended when the octopus is threatened. Nesis (1987) and Orsi-Relini (2009) also noted that both segments of the web and dorsal arms can be detached to protect the mass of embryos, which are brooded on the web until hatching (Portmann 1952). Figure 2G–I shows one segment of the web detached and with a chromatic pattern consisting of a large, round spot encircled by minor shapes, which is typical of *T. violaceus* (Orsi-Relini 2009).

**Figure 6.**
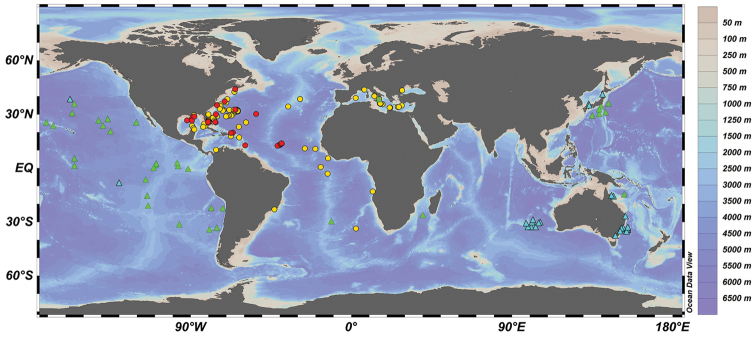
Observed distribution of coherent available records of *Tremoctopus violaceus* (dots) and *T. gracilis* (triangles) based on material examined by Thomas (1977) (red dots and green triangles), records contained in OBIS (2020) database and published records by Quetglas et al. (2013) and Almeida-Tubino et al. (2010) (yellow dots and blue triangles). Map prepared using Ocean Data View software (Schlitzer 2016).

The genetic analysis of the mtDNA 16S region revealed two important results. The phylogenetic inference confirms the identity of the Gulf of Mexico specimen as *T. violaceus*, i.e., within the same clade containing KY649286, MN435565, and AJ25276 (100 bootstrap support). The 6% genetic distance between analysed specimens suggests that the Gulf of Mexico and Pacific Ocean specimens belong to different species, with the Pacific Ocean species corresponding to *T. gracilis*. The average calculated interspecific genetic distance value for the mtDNA16S for cephalopods is 7.1% (range 1.3–12.7%) and for intraspecific genetic distances it is 0.5% (range 0.0–2.7%) (Dai et al. 2012). According to several authors (Thomas 1977; Almeida-Tubino et al. 2010; Quetglas et al. 2013; Finn 2014) and most of the records in OBIS (2020), *T. violaceus* occurs only in the Atlantic Ocean, whereas *T. gracilis* inhabits the Pacific and Indian oceans (Fig. 6). Therefore, it is likely that the octopods from the Pacific were misidentified and are in fact *T. gracilis*. The 16S marker is more variable than COI (Strugnell and Lindgren 2007) and is therefore a reliable marker for identifying species (Dai et al. 2012; Pliego-Cardenas et al. 2014, 2016; Flores-Valle et al. 2018). According to Chiu et al. (2018), *T. violaceus* is basal in the phylogenetic tree of Octopoda.

Data on the occurrence of *T. violaceus* are sporadic, with fewer than 350 records during the last hundred years, and many of these are from the Western Central Atlantic and the Mediterranean Sea (Fig. 6), which is consistent with the distribution of this taxon, as determined by Thomas (1977). From the Gulf of Mexico, available data are concentrated in the eastern portion of the Gulf (Fig. 1), near the influence of the Loop Current which exchanges water between the Caribbean Sea and the Eastern Seaboard. As far as we know, the specimen reported in this study is the first record of *T. violaceussensu stricto* from the southwestern Gulf of Mexico.

Finally, the molecular evidence of the new 16S haplotype of *T. violaceus* undoubtedly separates it from the few available haplotypes of *Tremoctopus gracilis* of the Pacific. More studies, with consideration to inter- and intraspecific geographic dispersion, is required to fully solve the molecular phylogeny of the genus.

## Supplementary Material

XML Treatment for Tremoctopus violaceus

## Supplementary materials

Supplementary material 1 Data resources Data type occurences, genbank accession numbers, hyperlink of molecular sequences Explanation note We presented the biological material examined in this study. Also we presented a table with specimens name, catalog number, GenBank accession numbers and hyperlink of molecular sequences used in this study. https://binary.pensoft.net/file/501550This dataset is made available under the Open Database License (http://opendatacommons.org/licenses/odbl/1.0/). The Open Database License (ODbL) is a license agreement intended to allow users to freely share, modify, and use this Dataset while maintaining this same freedom for others, provided that the original source and author(s) are credited.María de Lourdes Jiménez-Badillo, César Meiners-Mandujano, Gabriela Galindo-Cortes, Piedad Morillo-Velarde, Roberto González-Gómez, Irene de los Angeles Barriga-Sosa, Ricardo Pliego-Cárdenas
