# Supplementary material for: The first record of Tremoctopus violaceussensu stricto Delle Chiaje,1830 in southwestern Gulf of Mexico gives a hint of the taxonomic status of Tremoctopus gracilis
Source: Zookeys. 2021 Jan 26;1012:55–69. doi: 10.3897/zookeys.1012.55718 (PMC7854555; doi:10.3897/zookeys.1012.55718)
Supplement: Supplementary material 1 — Data resources [file zookeys-1012-055-s001.docx]

Data resources

Material examined

MEXICO • 1 female, 135 mm ML; Southwestern Gulf of Mexico, Veracruz, Antón Lizardo; 19^o^ 03’24’’ N, 95 ^o^ 59’17’’ W; 20 July 2019; Jiménez-Badillo, L; recovered alive by fishermen; GenBank: MT271737; Specimen code CNMO 8042.

Specimens name, catalog number, GenBank accession numbers and hyperlink of molecular sequences used in this study.

| Species | Specimen Catalog # | GenBank # | Hyperlink |
| --- | --- | --- | --- |
|  |  | 16S |  |
| *Tremoctopus violaceus* | CNMO 8042 | MT271737 | <https://www.ncbi.nlm.nih.gov/nuccore/MT271737> |
| \| *Tremoctopus violaceus* \| \| --- \| | NA | KY649286 | <https://www.ncbi.nlm.nih.gov/nuccore/KY649286> |
| *Tremoctopus violaceus* | \| NA \| \| --- \| | MN435565 | <https://www.ncbi.nlm.nih.gov/nuccore/MN435565> |
| \| *Tremoctopus violaceus* \| \| --- \| | \| UMML 31.312 \| \| --- \| | AJ252767 | <https://www.ncbi.nlm.nih.gov/nuccore/AJ252767> |
| *Haliphron atlanticus* | USNM1132848 | AY616971 | <https://www.ncbi.nlm.nih.gov/nuccore/AY616971> |
| *Argonauta nodosus* | NA | AY545104 | <https://www.ncbi.nlm.nih.gov/nuccore/AY545104> |
| *Argonauta hians* | NA | KY649285 | <https://www.ncbi.nlm.nih.gov/nuccore/KY649285> |
| *Argonauta argo* | NA | AB191108 | <https://www.ncbi.nlm.nih.gov/nuccore/AB191108> |
| *Ocythoe tuberculata* | NA | GU288520 | <https://www.ncbi.nlm.nih.gov/nuccore/GU288520> |
|  |  |  |  |
